# Comparative analysis of single-cell transcriptome reveals heterogeneity and commonality in the immune microenvironment of colorectal cancer and inflammatory bowel disease

**DOI:** 10.3389/fimmu.2024.1356075

**Published:** 2024-03-11

**Authors:** Hongchao Lv, Yu Mu, Chen Zhang, Meiqi Zhao, Ping Jiang, Shan Xiao, Haiming Sun, Nan Wu, Donglin Sun, Yan Jin

**Affiliations:** ^1^ Laboratory of Medical Genetics, Harbin Medical University, Harbin, Heilongjiang, China; ^2^ Key Laboratory of Preservation of Human Genetic Resources and Disease Control in China (Harbin Medical University), Ministry of Education, Harbin, Heilongjiang, China; ^3^ College of Bioinformatics Science and Technology, Harbin Medical University, Harbin, Heilongjiang, China

**Keywords:** comparative analysis, heterogeneity, commonality, colorectal cancer, inflammatory bowel disease, aging and inflammation

## Abstract

**Background:**

During aging, chronic inflammation can promote tumor development and metastasis. Patients with chronic inflammatory bowel diseases (IBD) are at an increased risk of developing colorectal cancer (CRC). However, the molecular mechanism underlying is still unclear.

**Methods:**

We conducted a large-scale single-cell sequencing analysis comprising 432,314 single cells from 92 CRC and 24 IBD patients. The analysis focused on the heterogeneity and commonality of CRC and IBD with respect to immune cell landscape, cellular communication, aging and inflammatory response, and Meta programs.

**Results:**

The CRC and IBD had significantly different propensities in terms of cell proportions, differential genes and their functions, and cellular communication. The progression of CRC was mainly associated with epithelial cells, fibroblasts, and monocyte-macrophages, which displayed pronounced metabolic functions. In particular, monocyte-macrophages were enriched for the aging and inflammation-associated NF-κB pathway. And IBD was enriched in immune-related functions with B cells and T cells. Cellular communication analysis in CRC samples displayed an increase in MIF signaling from epithelial cells to T cells, and an increase in the efferent signal of senescence-associated SPP1 signaling from monocyte-macrophages. Notably, we also found some commonalities between CRC and IBD. The efferent and afferent signals showed that the pro-inflammatory cytokine played an important role. And the activity of aging and inflammatory response with AUCell analysis also showed a high degree of commonality. Furthermore, using the Meta programs (MPs) with the NMF algorithm, we found that the CRC non-malignant cells shared a substantial proportion of the MP genes with CRC malignant cells (68% overlap) and IBD epithelial cells (52% overlap), respectively. And it was extensively involved in functions of cell cycle and immune response, revealing its dual properties of inflammation and cancer. In addition, CRC malignant and non-malignant cells were enriched for the senescence-related cell cycle G2M phase transition and the p53 signaling pathway.

**Conclusion:**

Our study highlights the characteristics of aging, inflammation and tumor in CRC and IBD at the single-cell level, and the dual property of inflammation-cancer in CRC non-malignant cells may provide a more up-to-date understanding of disease transformation.

## 1 Introduction

Colorectal cancer (CRC) is a malignant tumor with the third cause of new cancer cases and cancer deaths worldwide (1). During aging, chronic inflammation can affect the cells of the tumor microenvironment (TME), such as fibroblasts and immune cells (2). It can promote tumor progression and metastasis (3). Senescence prevents the proliferation of potentially cancerous cells, and acts as a potent anti-tumor mechanism. For example, interactions between the *p53/ARF* and *RB/p16* tumor suppressor pathways can block the cell cycle and play a central role in regulating senescence (4). The senescence-associated secretory phenotype induction relies on the activation of the inflammatory TFs *NF-κB*, a chronic DNA damage response (5). As a chronic inflammatory disorder, inflammatory bowel disease (IBD), which includes ulcerative colitis (UC) and Crohn’s disease (CD), increased the risk of developing CRC (6). IBD-related CRC is responsible for approximately 2% of annual mortality in CRC patients, and has a 5-year survival rate of 50%. It also affected patients at a younger age compared to sporadic CRC (7). Although there are many differences between IBD-related colorectal cancer and sporadic CRC, a study by Shailja C. Shah et al. suggested that colitis-associated CRC shares many molecular similarities with sporadic CRC (8). The factors generated by the host immune response may contribute to the inflammatory, aging and carcinogenic processes. Therefore, it is important to explore the potential differences and similarities between CRC and IBD as an immediate and urgent objective.

Single-cell RNA sequencing (scRNA-Seq) technology has the potential to unravel the diversity of cell states and the heterogeneity of cell populations (9). It serves as a powerful tool for investigating heterogeneous tissues, such as the tumor microenvironment (TME) (10). Extensive research has been conducted on the heterogeneity of immune cells in intestinal diseases, particularly tumors. For example, Zhang L et al.’s study utilized scRNA-seq analysis on colorectal cancer (CRC) patients and identified specific subsets of macrophages and conventional dendritic cells (cDCs) as key mediators in the TME (11). Pelka K et al.’s research discovered a mismatch repair-deficient (MMRd) enriched immune hub in the MMRd CRC individuals, with activated T cells together with malignant and myeloid cells expressing T cell-attracting chemokines (12). Similarly, a study on colonic mucosa and peripheral blood mononuclear cells from ulcerative colitis (UC) or Crohn’s disease (CD) patients revealed increased abundances of HLA-DR+CD38+ T cells, CXCR3+ plasmablasts, IL1B+ macrophages, and monocytes in the colonic mucosa samples from IBD patients (13). However, a clear characterization of the differences and similarities at the single-cell level between CRC and IBD is lacking.

In this study, we performed a comprehensive analysis of single cell transcriptomes in both CRC and IBD, comprising a total of 432,314 cells, which contained 92 CRC patients, 24 IBD patients, as well as 59 normal samples. Our analytical approach included the following key components: 1) providing an landscape by enumerating proportions, the differentially expressed genes (DEGs), and their functions for each cell subpopulation in both CRC and IBD; 2) comparing the intercellular communication mediated by receptor-ligand pairs to elucidate potential mechanisms of signaling interactions between cells; 3) evaluating the activity of aging and inflammation between CRC and IBD; 4) defining gene sets of Meta programs (MPs) as a means to capture the pattern of intra-disease heterogeneity. The overall objective of this study is to generate novel insights into the intra-disease pathogenesis of CRC and IBD.

## 2 Methods and materials

### 2.1 scRNA-seq data processing and cell type identification

By screening the Gene Expression Omnibus (GEO) and the Arrayexpress databases, we obtained scRNA-seq data from eight studies on colorectal cancer (GSE132257, GSE132465, GSE200997, GSE166555, GSE188711, GSE144735, GSE161277, EMTAB.8107), and five studies on inflammatory bowel disease (GSE150115, GSE164985, GSE182270, GSE184291, GSE134809). We constructed a single-cell metadata profile of intestinal disorders. All scRNA-seq data were obtained from published studies with raw counts for 10X Genomics, and the sample information is described in **Supplementary Table 1**. To perform batch correction, data integration, and quality control, we utilized the R packages Seurat v4 and Harmony v0.1.1 on a total of 175 single-cell samples from thirteen studies (14, 15). We filtered out genes that were detected in 5 or fewer cells, as well as cells with an expressed gene count lower than 2% or greater than 98% (16). The samples were then normalized using logarithmic normalization. We further identified the top 30 principal components using principal component analysis (PCA) on the top 3000 highly variable genes. Clustering was performed using the FindNeighbors and FindClusters functions (resolution = 0.8), and all cells were classified into 45 clusters using the uniform manifold approximation and projection (UMAP) algorithm (17). Each cell cluster was annotated with well-known cell-type specific markers (18–20). Additionally, we employed the FindAllMarkers and FindMarkers functions in Seurat to identify the differentially expressed genes (DEGs) for each cell type based on the non-parametric Wilcoxon rank-sum test. We then combined the DEGs with the CellMarker website for further manual annotation of cell types (21).

### 2.2 Identification of malignant cells in colorectal cancer

Considering the presence of tumor heterogeneity, we integrated and used two methods, namely Copy Number Karyotyping of Aneuploid Tumors (CopyKAT) and Single Cell Variational Aneuploidy Analysis (SCEVAN), to distinguish malignant cells from non-malignant epithelial cells (22, 23). CopyKAT is an integrative Bayesian approach with hierarchical clustering to quantify genomic copy number profiles and define clonal substructure from high-throughput scRNA-seq data. It can be used to identify tumor cells in the TME and is implemented using the R package “copykat” (22). On the other hand, SCEVAN is a fast variational algorithm for deconvoluting the clonal substructure of tumors from scRNA-seq data. It assumes that all cells within a given copy number clone share the same breakpoints. Using the R package SCEVAN, it can automatically and accurately discriminate between malignant and non-malignant cells (23). Here, we assigned the epithelial cells of CRC patients as malignant and non-malignant cells (22, 23) using the R packages copykat and SCEVAN. Through the integration of both approaches, we successfully defined 32,387 malignant cells and 23,805 non-malignant cells with high confidence.

### 2.3 Defining robust NMF programs and Meta programs

We performed the non-negative matrix factorization (NMF) process separately for each of the included studies to generate a program that captures the intercellular heterogeneity. Here we used the computational scheme proposed by Avishai Gavish et al. to obtain robust NMF programs (24). 1) NMF was run with different parameter values (k=4, 5, 6, 7, 8, 9), generating 39 programs for each study. 2) Each NMF program was used to synthesize the top 50 genes, selected by their coefficients. 3) The robust NMF programs were then selected, using different values of k to obtain programs with at least 70% overlap (35 out of 50 genes) and more than 20% similarity across studies.

Finally, the robust NMF programs were clustered based on Jaccard similarity to identify a new cluster. We compared each robust NMF program with all other robust NMF programs (>10 genes), assessing the degree of genetic overlap. The NMF program with the highest gene overlap was designated as the founder NMF program. We repeated this process by searching for the next program with maximal overlap (>10 genes) with the cluster and adding it to the cluster until no additional NMF program could be added. We denoted this cluster as a Meta program (MP), and defined the top 50 genes as the gene set most commonly shared between programs from that cluster (24). The part of the code were from Avishai Gavish et al.`s research (available in https://github.com/tiroshlab/3ca). We performed the non-negative matrix factorization (NMF) process separately for each of the included studies to generate a program that captures the intercellular heterogeneity.

### 2.4 Gene set enrichment analysis, Functional enrichment analysis, and AUCell analysis

We utilized the R package msigdbr V7.5.1 to retrieve gene sets from the Human Molecular Signatures Database (MSigDB). The MSigDB includes Hallmark gene sets, Gene Ontology gene sets for biological processes (GOBP), molecular functions (GOMF), and cellular components (GOCC), canonical pathways gene sets, and so on (25). Gene set enrichment analysis was conducted on the DEGs identified for each cell type between the patients and normal samples. Additionally, we assessed the enrichment of marker genes with Gene Ontology terms (C5:BP/CC/MF) and pathway enrichment analysis using a hypergeometric test (FDR-adjusted P<0.05 was considered significant). All of the above analyses are implemented using the R package clusterprofiler (26). AUCell is an approach to determine the activity of specified gene sets on single cell RNA-seq data (27). The area under the curve (AUC) is used to calculate whether the input gene set is enriched within the expressed genes for each cell.

### 2.5 Cell-cell communication analysis

A toolkit CellChat used to quantitatively infer and analyze intercellular communication networks from scRNA-seq data (28). It is based on the database of interactions among ligands, receptors and their cofactors. It can predict key signaling inputs and outputs for cells, and the functional coordination between cells and signals using network analysis and pattern recognition approaches. The *P* value< 0.05 was filtered.

### 2.6 Software packages

Data analysis was performed in R (versions 4.1.1 and 4.3.0) with the following packages: Seurat (version 4.3.0.1), harmony (version 0.1.1), monocle3 (version 1.3.1), AUCell (version 1.24.0), NMF (version 0.26), msigdbr (version 7.5.1), clusterprofiler (version 4.8.2), CellChat (version 1.6.1), SCEVAN (version 1.0.1), copykat (version 1.1.0), ggplot2 (version 3.4.3), and pheatmap (version 1.0.12).

## 3 Results

### 3.1 Epithelial cells increased with the progression of CRC whereas B cells were more concentrated in IBD patients

**Figure 1:**
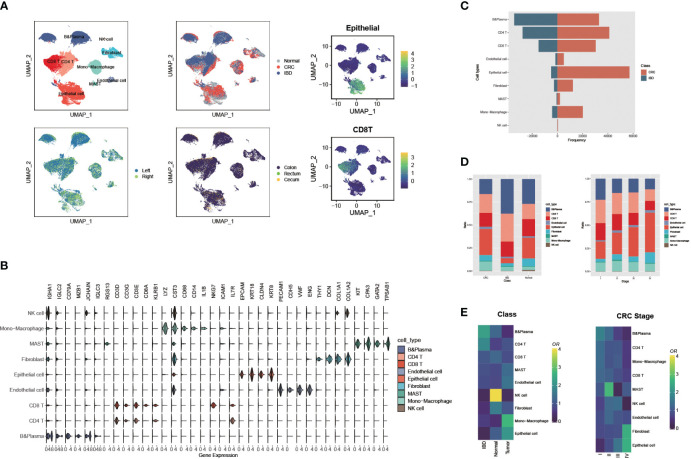
Large-scale single cell landscapes of colorectal cancer and inflammatory bowel disease. **(A)** Uniform Manifold Approximation and Projection (UMAP) plot showing the immune landscape of CRC and IBD identified by integrated analysis of 175 samples, colored by nine cell clusters, disease type, position, organism, and gene scores (Epithelial and CD8 T cells). **(B)** The expressions of marker genes or characteristic genes for each cell type. **(C)** Comparison of the frequency of nine cell types recognized in CRC and IBD. **(D)** Comparison of the proportions of nine cell types in CRC and IBD, and different CRC stages. **(E)** Tissue preference of each cluster estimated by Ro/e in CRC and IBD, and different CRC stages.

We integrated scRNA-seq data from eight colorectal cancer studies (92 tumor samples, 49 para-carcinoma tissues), and five inflammatory bowel disease studies (24 IBD samples and 10 normal samples), and constructed a scRNA-seq metadata profile of the intestinal disorders (**Supplementary Table 1**, detail see Methods and Materials). To ensure comparability between IBD and CRC in scRNA-seq data, we used the R packages Seurat and harmony for integration and quality control. As a result, we obtained 432,314 cells, which were then classified into 45 clusters. Based on the previously reported marker genes, as well as the human cell marker genes from the CellMarker website, we identified nine major cell types. Then the UMAP plots were shown in **Figure 1A** and **Supplementary Figure 1A**, which were colored by nine cell clusters, disease type, position, organism, and gene scores with cell type markers that calculated by function AddModuleScore. The marker genes or characteristic genes for each cell type are as following: B cells (*CD79A, MZB1, JCHAIN, IGLC3, RGS13*), plasma cells (*IGHA1, IGLC2*), CD4+ T cells (*CD3D, CD3G, CD3E*), CD8+ T cells (*KLRB1, CD8A*), natural killer (NK) cells (*NKG7, ICAM1, IL7R*), monocyte-macrophages (*LYZ, CST3, CD14, CD68, IL1B*), endothelial cells (*CDH5, PECAM1, VWF, ENG*), fibroblasts (*DCN, THY1, COL1A, COL1A2*), MAST cells (*KIT, CPA3, GATA2, TPSAB1*), and epithelial cells (*EPCAM, KRT18, CLDN4,KRT8*) (**Figure 1B**).

Comparing the frequency and proportions of the nine cell types recognized in CRC and IBD, significant differences can be observed. The proportions of epithelial cells and monocyte-macrophages were remarkably higher in CRC patients, whereas the B cells and CD4 T cells were more abundant in IBD (**Figures 1C, D**). Interestingly, the distribution of cells at different stages of CRC also showed a decrease in the proportion of immune cells as the disease progresses, while epithelial cells increased in advanced stage CRC patients (**Figure 1D**). Notably, this distribution trend was discordant in the cell proportions of individual samples, most likely due to the high prevalence of dropouts associated with single-cell RNA sequencing (**Supplementary Figure 1B**). To account for the possibility of sampling bias in the integrated samples for CRC and IBD, we also compared the tissue distribution of cells in IBD, CRC patients and normal tissues using a quantitative indicator of the tissue preference called the Ro/e metric (29). The Ro/e metric compares the ratio of the number of observed cells to the number of expected cells using Fisher’s exact test to quantify the degree of tissue preference of each sub-population. We also found that the B cells, CD4T cells tend to be distributed in IBD, while the epithelial cells, fibroblasts, and monocyte-macrophages tend to aggregate in CRC patients (**Figure 1E**). In addition, the proportion of B cells, CD4 and CD8 T cells decreases with CRC progression, and epithelial cells and fibroblasts become increasingly enriched. Frede A et al. study pointed out that B cells were the major cell type in the healing colon and IFN-induced expansion of B cell subpopulations reduced the interaction between stromal and epithelial cells, and thus affected intestinal mucosal healing (30). In contrast, Non-malignant cells in the tumor microenvironment, including fibroblasts, immune cells, and endothelial cells, contribute to tumor progression through complex interactions with cancer cells, such as fibroblasts support tumor growth and metastasis and regulate inflammatory responses and cell proliferation in tumor tissues (31, 32).

### 3.2 CRC showed significant activity increases in metabolism-related functions while IBD displayed powerful associations with immune-related functions

**Figure 2:**
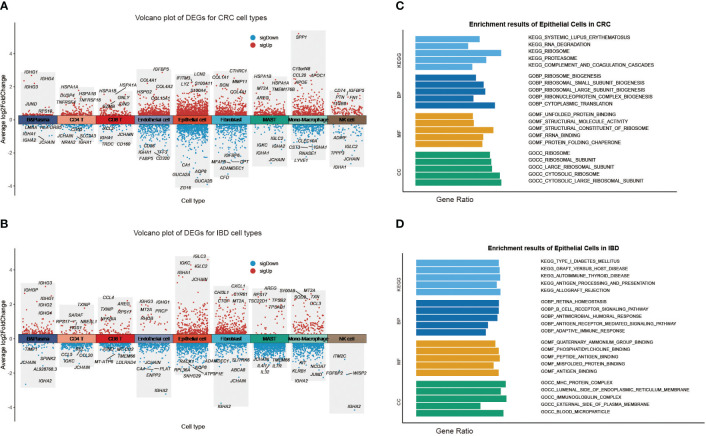
Heterogeneity of each cell type in CRC and IBD. **(A)** Volcano plot of DEGs for each CRC cell type. **(B)** Volcano plot of DEGs for each IBD cell type. Gene set enrichment analysis (GSEA) results of epithelial Cells **(C)** in CRC; **(D)** in IBD.

To further investigate the intra-disease heterogeneity of each cell state encompassed by CRC and IBD, we focused on the variability of case-control samples of each cell subpopulation (reflecting disease identity), and explored their functions. Using Seurat’s FindAllMarkers function and Wilcoxon rank sum test, we identified the DEGs for each cell subpopulation in CRC and IBD, separately. Excepting for immune cell-specific marker genes, there was a markedly different predisposition for the DEGs obtained (**Figures 2A, B**; **Supplementary Figure 2A**). We further explored the functional heterogeneity of epithelial cells in two diseases, and performed gene set enrichment analysis (GSEA) using the signatures from MsigDB database (**Figures 2C, D**). Pathway enrichment analysis of epithelial cells showed that the DEGs in CRC displayed increased activity in metabolism-related pathways such as ribosomes, proteasomes, fatty acid metabolism, and nitrogen metabolism, along with T-cell receptor signaling pathways. However in IBD, there was a significant increase in activity observed in immune-related pathways such as type 1 diabetes mellitus, graft-versus-host disease, autoimmune thyroid disease, and antigen processing and presentation. GO functional enrichment analysis of epithelial cells showed that CRC-related genes were more involved in functional nodes associated with ribosomes and protein synthesis, while IBD-related genes were associated with adaptive immune responses, signaling pathways, antigen binding and other immune functions (**Figures 2C, D**). These findings reflect significantly different tendencies in the molecular characterization of CRC and IBD. To account for the organizational heterogeneity, we also performed differentially expressed gene (DEG) analysis and pathway enrichment analysis on epithelial cells, CD4 T cells, CD8 T cells and B cells from the colon and rectum (**Supplementary Table 2**). The results showed that there was significant variation between cell proportions and DEGs, but a high degree of commonality was found when pathway enrichment analysis of DEGs was performed (**Supplementary Figures 3A, B**).

**Figure 3:**
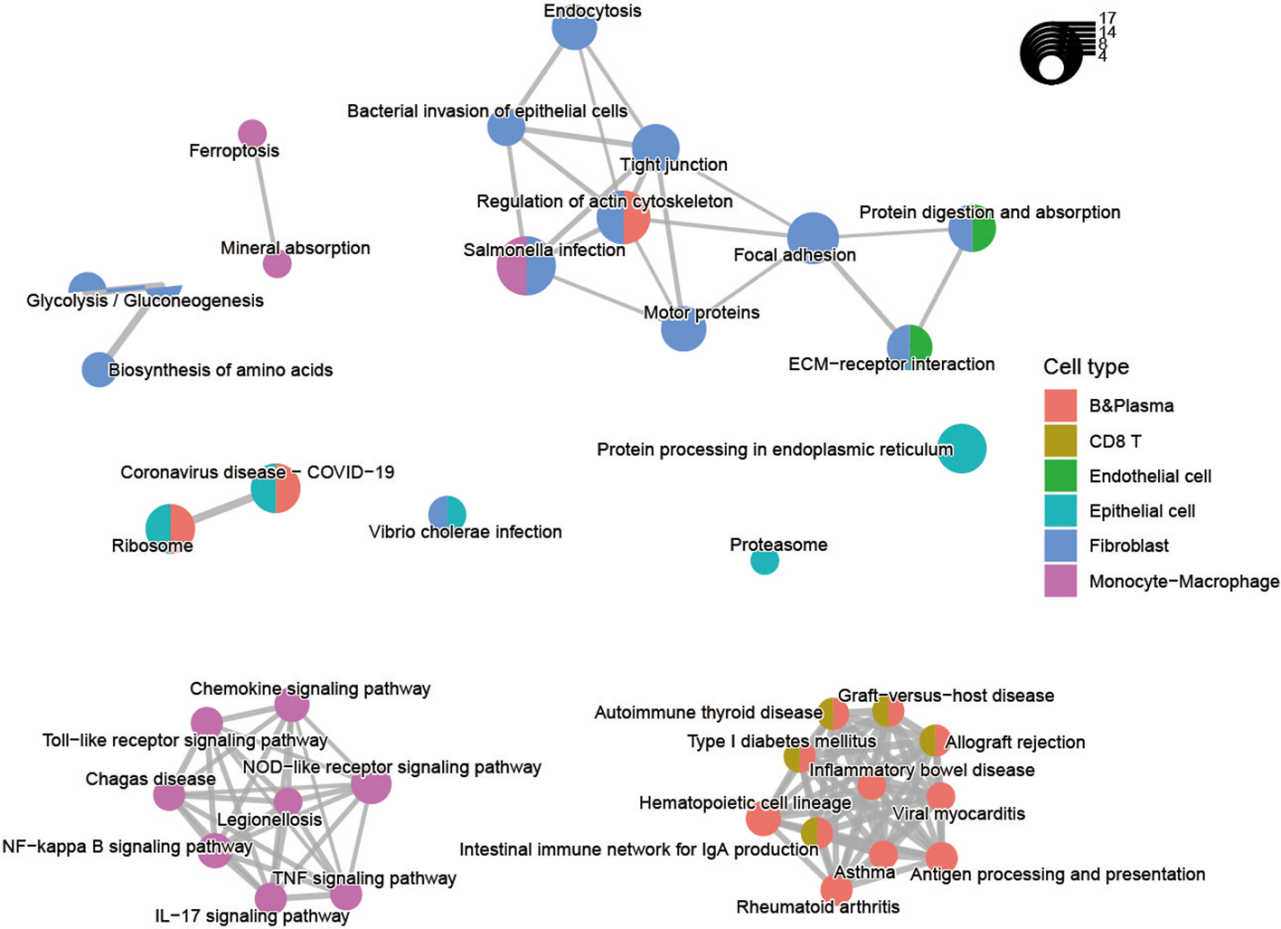
Pathway enrichment analysis of the up-regulated differentially expressed genes for nine cell types shared by CRC and IBD.

We subsequently investigated the function of the up-regulated DEGs shared in two diseases (**Figure 3**; **Supplementary Figure 2B**). The results displayed that epithelial cells were primarily involved in ribosomal, proteasomal and endoplasmic reticulum protein processing, as well as infectious diseases. The B cells and T cells were mainly enriched in immune disease pathways such as rheumatoid arthritis, autoimmune thyroid disease, and inflammatory bowel disease. These cells also played a role in the immune system, the intestinal immune network for IgA production, and antigen processing and presentation. And the stromal and endothelial cells were found to be rich in protein functions, including focal adhesion, regulation of the actin cytoskeleton, *ECM*-receptor interaction, proteoglycans in cancer, protein digestion and absorption. The monocyte-macrophages were associated with various tumor-related signaling pathways, such as *NF-kappa B* signaling pathway, *TNF* signaling pathway, *NOD*-like receptor signaling pathway, Toll-like receptor signaling pathway, Chemokine signaling pathway, *IL-17* signaling pathway. In particular, the pathway activation of NF-kappa B has a causal role in promoting senescence, and the association of them have response to chemotherapy (33, 34). Through NF-kappa B in epidermal cells, aberrant IL-17 signaling during ageing impairs homeostatic functions, and promotes an inflammatory state (35). The functional annotations of the biological process GO also demonstrated that each cell had a distinct role in the development of diseases. For instance, epithelial cells are mainly associated with metabolism-related functions, whereas monocytes and macrophages respond primarily to biotic stimuli (**Figure 3**; **Supplementary Figure 4**). The results revealed the variation in the intercellular molecular functions between different cell types.

### 3.3 CRC showed greater intensity of communication in epithelial cells and fibroblasts but IBD mainly in immune cells

**Figure 4:**
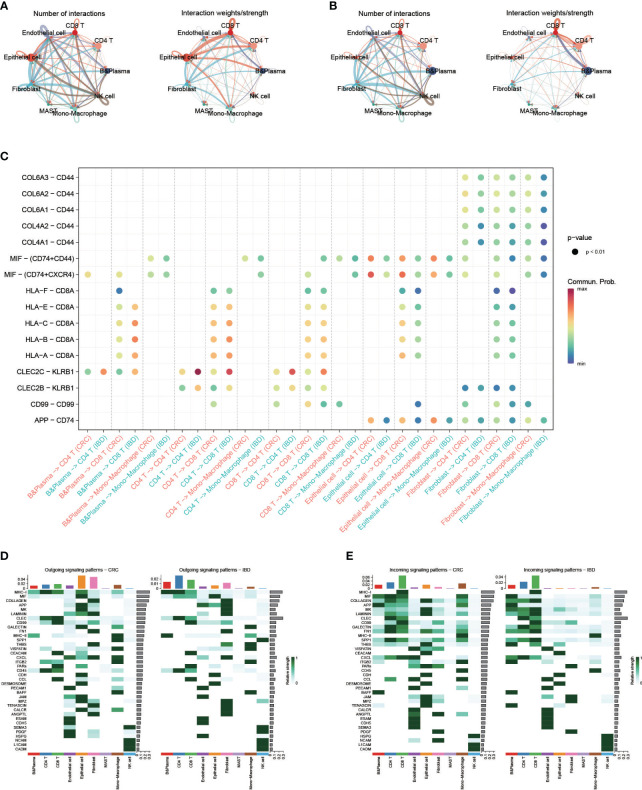
Single-cell transcriptional analysis reveals the cell communication in CRC and IBD. **(A)** Analysis of the number of interactions and interaction strength between different cell types in CRC samples. **(B)** Analysis of the number of interactions and interaction strength between different cell types in IBD samples. **(C)** Identification of signaling by comparing the communication probabilities mediated by ligand-receptor pairs from macrophages to other cell types in CRC and IBD samples. **(D, E)** Overview of the outgoing signaling and incoming signaling in CRC and IBD samples.

To explore the characteristics of cellular interactions in CRC and IBD, we analyzed cell communication using the CellChat package. The results revealed significant differences in cellular communication between the two diseases. In CRC, there were greater intensity of communications observed in epithelial cells and fibroblasts, which exhibited close connections to immune cells and monocyte-macrophages. On the other hand, in IBD, the communication was primarily between B cells and CD8 T cells, as well as CD4 and CD8 T cells (**Figures 4A, B**). We compared the changes in ligand-receptor pairs across cell types of the two diseases (**Figure 4C**). In CRC samples, an increase in *MIF* signaling (e.g. *MIF*-*(CD74+CD44)*, *MIF-(CD74+CXCR4)*) from epithelial cells to CD4T and CD8T cells was observed. As a pro-inflammatory cytokine, macrophage migration inhibitory factor (*MIF*) accelerated deleterious inflammation and promoted cancer metastasis and progression (36). *MIF* interacted with the surface *CD74*, inducing its phosphorylation and the recruitment of *CD44*, ultimately leading to *ERK1/2* phosphorylation (37). In contrast, signaling in the IBD samples increased between CD4 and CD8 T cells, as well as from B cells to T cells (e.g. *CLEC2C-KLRB1*, *HLA-B-CD8*A, *HLA-C-CD8A*). Furthermore, we outlined the efferent and afferent signalling in CRC and IBD samples (**Figures 4D, E**). In CRC, the major efferent signals for epithelial cells included MIF, APP and MK signals, while for monocyte-macrophages included MHC-II, CXCL and SPP1 signals, which the high expression of SPP1 in macrophages having strong senescence-associated secretory phenotype (SASP) features (38). Conversely, in IBD samples, the primary efferent signals for B cells and T cells were *MHC-I*, *MHC-II*, *CLEC* and *CD99* signals. Afferent signals were mainly concentrated on immune cells in both CRC and IBD samples, with the main signals being *MHC-I*, *MIF*, *COLAGEN*, *APP*, *CLEC*, *GALECYIN*, *FN1*, *SPP1* and *CXCL* signals. These results demonstrate that the pro-inflammatory cytokine plays a role in the development of inflammation and cancer, particularly in malignant transformation, invasion and metastasis of cancer.

### 3.4 AUCell analysis showed CRC and IBD had a high degree of commonality in aging and inflammatory response

**Figure 5:**
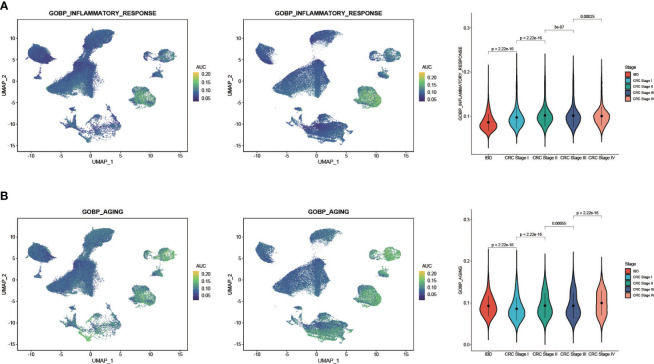
AUCell analysis of the regulation of aging and inflammatory in CRC and IBD. **(A)** GOBP_AGING scored per cell by AUCell among nine cell subtypes. **(B)** GOBP_INFLAMMATORY_RESPONSE scored per cell by AUCell among nine cell subtypes. The yellow dots indicate a strong activity, whereas dark blue dots indicate a weak activity.

We obtained two classical gene sets from MsigDB database, GOBP_AGING and GOBP_INFLAMMATORY_RESPONSE, to compare the relationships of aging and inflammatory responses between CRC and IBD. We then extracted CRC patients with disease stage, and then obtained a total of 61 CRC and IBD samples with 240,501 cells. We re-clustered these samples and used the AUCell scores to assess the activity of the gene set in each cell subtype. The results show that inflammatory response and aging functions had a high degree of commonality in both CRC and IBD (**Figures 5A, B**). Function of inflammatory response was enhanced mainly in the monocyte-macrophage subset, and aging function was more active in monocyte-macrophages, fibroblasts, and epithelial cells. In particular, we also observed a significant enhanced activity of inflammatory response and aging with the progression of CRC, revealed a tight relationship between CRC and IBD (**Figures 5A, B**).

### 3.5 CRC non-malignant cells shared a substantial proportion of Meta program genes with CRC malignant cells and IBD epithelial cells

**Figure 6:**
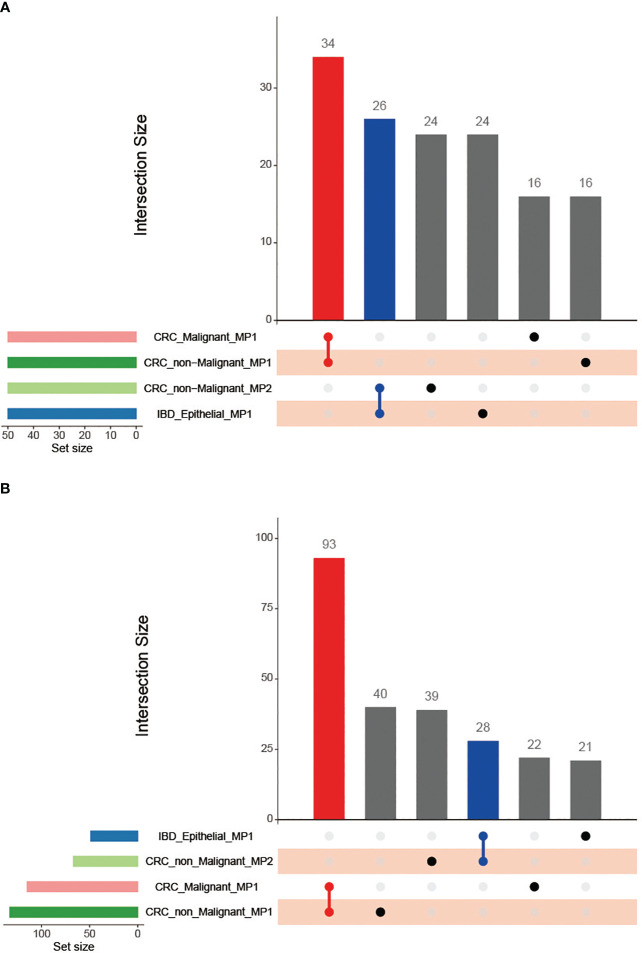
Venn diagram of the Meta programs and their functions recognized in CRC malignant cells, CRC non-malignant cells, and IBD epithelial cells using the top 50 genes. **(A)** Comparison of the number of Meta programs; **(B)** Comparison of the MP-related functional categories.

We further compared the heterogeneous characterization of epithelial cells using the NMF method. First, CRC epithelial cells were assigned into 32,387 high-confidence malignant cells and 23,805 non-malignant cells using the R packages copykat and SCEVAN (**Supplementary Figure 5**). We then utilized robust NMF programs to characterize the CRC malignant cells, CRC non-malignant cells, and IBD epithelial cells, respectively (see Methods and Materials for details). Overall, 71 robust NMF programs were detected in all samples studied. Comparing the stable NMF programs in the three sample types, we observed that only CRC malignant cells and non-malignant cells had robust NMF programs with more than 70% overlapping genes. Furthermore, according to the fractions of shared top genes, we clustered the robust NMF programs and identified four MPs (**Figure 6A**), checking the top 50 genes as the common gene set between the programs (see Methods and Materials for details). Our analysis revealed that no genes were shared between CRC malignant cells and IBD epithelial cells (**Figure 6A**; **Supplementary Table 3**). However, CRC non-malignant epithelial cells shared a substantial proportion of the MP genes with both CRC malignant cells and IBD epithelial cells, with a 68% overlap (34 out of 50 genes) and a 52% overlap (26 out of 50 genes), respectively. This indicates a common molecular signature between inflammation and cancer, suggesting the potential presence of early transformation between them.

Similarly, we recognized 151 robust NMF programs among immune cells (B cells, CD4+ T cells, CD8+ T cells, and monocytes) in both CRC and IBD. Subsequently, we identified seven MPs in CRC immune cells and two MPs in IBD immune cells to compare intra-disease heterogeneity (**Supplementary Table 4**). The results revealed only a few shared genes among these MPs between CRC and IBD, illustrating distinct tendencies of immune cells across the two diseases. Of particular interest, we noted that the gene *S100A4* was common to both CRC immune cells (MP2) and IBD immune cells (MP2), contributing to colon inflammation and colitis-associated colon tumorigenesis (39). Several reports have demonstrated that *S100A4* enhanced colitis development by increasing the adherence of Citrobacter rodentium in intestinal epithelial cells (40). Moreover, increased *S100A4* expression significantly correlates with tumor angiogenesis, cell survival, motility, invasion, and metastasis (41). Additionally, our results showed that MP1 in CRC immune cells shares an overlap of 28 genes (56%) with both CRC malignant and non-malignant cells, suggesting a collaborative role in malignant tumor development (**Supplementary Table 4**). Notably, we observed the presence of pro-inflammatory cytokines (*IL1A*, *IL1B*, *IL6*), and chemokines (*CCL3*, *CXCL2*, *CXCL3*) in CRC immune cells (MP7). These molecules are downstream of *NF-κB* and have been shown to promote inflammation-driven neoplasia (42).

### 3.6 Functions of MPs in CRC non-malignant cells showed strong association with CRC malignant cells and IBD epithelial cells

**Figure 7:**
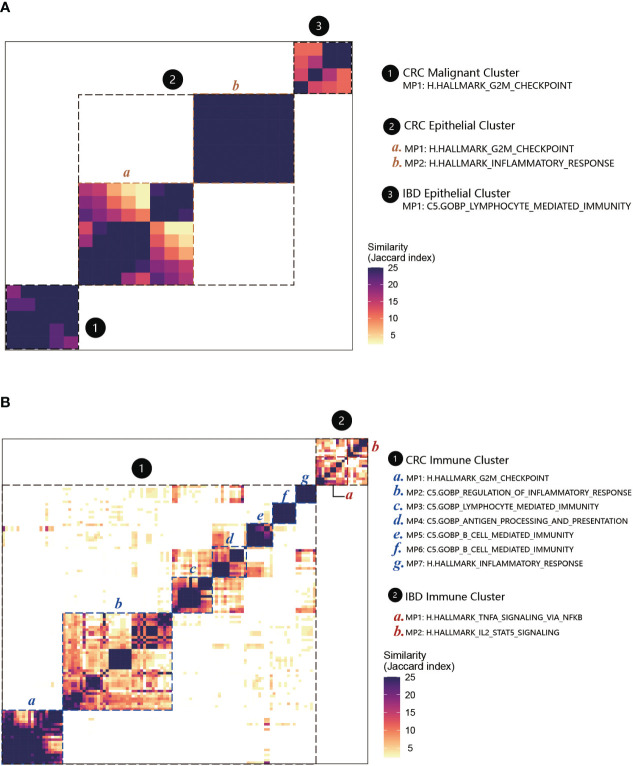
MPs and their functional annotations in CRC and IBD. **(A)** Heatmap showing Jaccard similarity indices for comparisons among 71 robust NMF programs of epithelial cells. The programs are ordered by clustering and grouped into four MPs with their related functions (marked by black dashed lines). The disease types are numbered and labeled. **(B)** Heatmap showing Jaccard similarity indices for comparisons among 151 robust NMF programs of immune cells. A total of seven MPs with their related functions were recognized in CRC immune cells, and two MPs were recognized in IBD immune cells (marked by black dashed lines).

We performed the functional enrichment analyses for MPs to compare the relationships among CRC malignant cells, CRC non-malignant cells, and IBD epithelial cells (**Figure 6B**; **Supplementary Table 5**). Similar as the MPs, we found that the functions of MPs in CRC non-malignant cells showed a strong association with CRC malignant cells and IBD epithelial cells. The CRC non-malignant cells shared 93 functional categories with CRC malignant cells, and shared 28 functional categories with IBD epithelial cells (**Figure 6B**). For MP in CRC malignant cells and the MP1 in CRC non-malignant cells, the functional features reflected primarily the role of the cell cycle in tumors, mainly containing G2M checkpoint (HALLMARK) and cell cycle G2M phase transition (GOBP) (**Figure 7A**; **Supplementary Table 5**). This finding was consistent with Avishai Gavish et al’s conclusion, who suggested that a significant portion of the heterogeneity observed in malignant cells already exists in the cells of origin (24). Furthermore, it is well known that a hallmark of cellular senescence is a stable cell cycle arrest in G1 or G2, which is mainly regulated by the *p53/ARF* and *RB/p16* pathways (43). Our study revealed the enrichment of the cell cycle G2M phase transition and the *p53* signaling pathway between two MPs. Interestingly, CRC non-malignant cells also exhibited another MP2 characterizing immune response, which shared high similarity with IBD epithelial cells (**Figures 6A**, **7A**; **Supplementary Table 5**). The common functional features of MPs included primary immunodeficiency (C2KEGG), T cell receptor signaling (C2KEGG), hematopoietic cell lineage (C2KEGG), allograft rejection (HALLMARK), monocyte differentiation (GOBP), and more. Studies have shown that the activation of the T cell receptor (TCR) promotes various signaling cascades, leading to T-cell proliferation, cytokine production, and differentiation into effector cells (44). Additionally, MP2 associated with CRC non-malignant cells exhibited inflammatory responses (HALLMARK) and lymphocyte apoptotic processes (GOBP). IBD epithelial cells also demonstrated the function of lymphocyte-mediated immunity (GOBP) (see **Supplementary Table 5**). These results underscored the extensive involvement of non-malignant epithelial tissue in the cell cycle and immune responses, and displayed their dual characteristics of inflammation and cancer in early non-malignant cells.

For the immune cells between CRC and IBD, we applied the above analyses to annotate the MPs (**Figure 7B**; **Supplementary Table 6**). Among the nine MPs recognized in CRC and IBD immune cells, several shared functional categories emerged. Notably, these encompassed *TNFA* signaling via *NF-κB* (HALLMARK) and *IL2-STAT5* signaling (HALLMARK), as well as primary immunodeficiency (C2.KEGG). Multiple studies have highlighted the significance of *NF-κB* activation in instigating acute and chronic inflammation, thereby establishing a link to the initiation and progression of gastrointestinal (GI) cancers through mechanisms involving chronic inflammation, cellular transformation, and proliferation (45, 46). In addition, the MPs in IBD immune cells were associated with apoptosis and receptor activity. And for CRC, they also involved in some immune cell-specific functions, such as granulocyte-specific C5.GOBP granulocyte migration, C5.GOBP granulocyte chemotaxis (MP7), B cell-specific C5.GOBP B cell mediated immunity and C5.GOBP B cell activation (MP5, MP6) (**Supplementary Table 6**). These findings underscore the pivotal role played by cancer-associated immune and inflammatory traits in these conditions. Furthermore, w e also recognized MP1 in CRC immune cells have overlap with CRC epithelial cells. The enrichment analyses also displayed some similar functions to the epithelial cells, such as involved H.HALLMARK G2M checkpoint (MP1) in the cell cycle, C5.GOBP regulation of inflammatory response (MP2), C5.GOBP lymphocyte mediated immunity (MP3), C5.GOBP antigen processing and presentation (MP4) (**Supplementary Table 6**).

## 4 Discussions

CRC and IBD are currently two of the common diseases in the intestinal tract, and IBD usually have an increased risk of developing CRC. Thus exploring the differences and connections between them has become a priority. Single-cell transcriptome data is a powerful tool for studying heterogeneous tissues, and helps to dissect the diversity of cell states and the heterogeneity of cell populations. Combined with 432,314 cells obtained from databases, we identified nine cell types of CRC and IBD. We then compared the patterns of intra-disease heterogeneity from cell proportions, differentially expressed genes and their functions, and cellular communication, separately. The results revealed significant disease specificity among nine cell types in CRC and IBD patients. Then using the NMF program to identify Meta programs based on scRNA-seq data to further characterize intercellular heterogeneity, we found that Meta programs and their functions in CRC malignant cells were extremely different from IBD epithelial cells, but the CRC non-malignant cells showed strong association with CRC malignant cells and IBD epithelial cells, respectively. In view of this situation, we performed trajectory analyses of IBD epithelial cells, CRC non-malignant cells and malignant cells using monocle3 software with default parameters. UMAP analysis revealed that IBD and CRC belonged to different clusters due to heterogeneity. The developmental trajectories of three epithelial cells were then inferred based on transcriptome changes. We selected CRC non-malignant cells in each cluster as the root point and performed trajectory analysis. As shown in the **Supplementary Figure 6**, the results of one cluster (at the bottom) showed that the developmental trajectories of the cells correlated with the malignant progression of the disease. Then by analyzing cell trajectories at different stages of the disease, we also found that the degree of disease progression was related to cell activity. Notably, the tumor epithelial cells in stage III showed higher levels of tumor cell activity than those in stage IV. It is a follow-up question that needs to be answered urgently whether this predicts a link to the cellular aging process. Additionally, MPs recognized between immune cells show strong functional similarity between IBD and CRC. Thus the results revealed strong similarities between IBD and CRC, both in non-malignant epithelial cells as well as immune cells, indicating a common immune mechanism of action between IBD and CRC. We identified pro-inflammatory cytokines (*IL1A*, *IL1B*, *IL6*), and chemokines (*CCL3*, *CXCL2*, *CXCL3*) that promote inflammation-promoted neoplasia. Specifically, MPs enriched for the functions of *TNFA* signaling via *NF-κB* become a strong support for inflammation and cancer transition.

Although our study characterized the intercellular heterogeneity of the two diseases from different perspectives, it has some limitations. All of the samples were retrospective and the results were mainly for the second mining. It was difficult to obtain the raw data, so we only performed the analysis for the single cell transcription profiles. We also lack the scRNA-seq data of IBD-associated CRC disease, combining the connections between IBD-associated CRC and sporadic CRC would be more definitive and reliable to compare the association between IBD and CRC. In conclusion, our study highlights the heterogeneity and commonality between CRC and IBD at the single-cell level, and the dual property of inflammation-cancer in CRC nonmalignant cells may provide a more up-to-date understanding of disease transformation.

## Data availability statement

The original contributions presented in the study are included in the article/**Supplementary Material.** Further inquiries can be directed to the corresponding authors.

## Author contributions

HL: Writing – original draft, Data curation, Software, Visualization, Writing – review & editing. YM: Investigation, Writing – original draft. CZ: Software, Visualization, Writing – original draft. MZ: Data curation, Investigation, Writing – original draft. PJ: Data curation, Validation, Writing – original draft. SX: Investigation, Validation, Writing – original draft. HS: Software, Validation, Writing – review & editing, Funding acquisition. DS: Funding acquisition, Investigation, Writing – review & editing. NW: Methodology, Validation, Writing – review & editing. YJ: Funding acquisition, Methodology, Writing – original draft, Writing – review & editing.

## Supplementary material

Supplementary Figure 1 Comparison of cell type distribution characteristics. **(A)** the UMAP plots of gene scores with cell type markers that calculated by function AddModuleScore. **(B)** The cell proportions of 10 individual samples between IBD and CRC.

Supplementary Figure 2 Differentially expressed genes (DEGs) of each cell subpopulation in CRC and IBD. **(A)** Venn diagram of DEGs in nine cell types. **(B)** Up-regulated DEGs among the six major cell types.

Supplementary Figure 3 The organizational heterogeneity among cell types. **(A)** Venn diagrams of differentially expressed gene, and **(B)** Venn diagram of pathway enrichment analysis on epithelial cells, CD4 T cells, CD8 T cells and B cells from the colon and rectum.

Supplementary Figure 4 GO biological process of the up-regulated differentially expressed genes for nine cell types shared by CRC and IBD.

Supplementary Figure 5 UMAP plots of the malignant cells identified by the R packages copykat and SCEVAN.

Supplementary Figure 6 the cell trajectory analysis results of IBD epithelial cells, CRC non-malignant cells and malignant cells. **(A)** The developmental trajectory in the pseudotime analysis. **(B)** The cell trajectory analysis results of malignant and non-malignant cells. **(C)** The cell trajectory analysis results of different stages.

Supplementary Table 1 Baseline Clinical characteristics of all samples in CRC and IBD.

Supplementary Table 2 Number of cell types in different organism parts.

Supplementary Table 3 MP genes in CRC malignant cells, CRC non-malignant cells and IBD epithelial cells.

Supplementary Table 4 MP genes in CRC and IBD immune cells.

Supplementary Table 5 Functional enrichment analyses for MPs in CRC malignant cells, CRC non-malignant cells, and IBD epithelial cells.

Supplementary Table 6 Functional enrichment analyses for MPs in CRC and IBD immune cells.
