# Supplementary figures and images for: Comparative analysis of single-cell transcriptome reveals heterogeneity and commonality in the immune microenvironment of colorectal cancer and inflammatory bowel disease
Source: Front Immunol. 2024 Mar 11;15:1356075. doi: 10.3389/fimmu.2024.1356075 (PMC10961339; doi:10.3389/fimmu.2024.1356075)

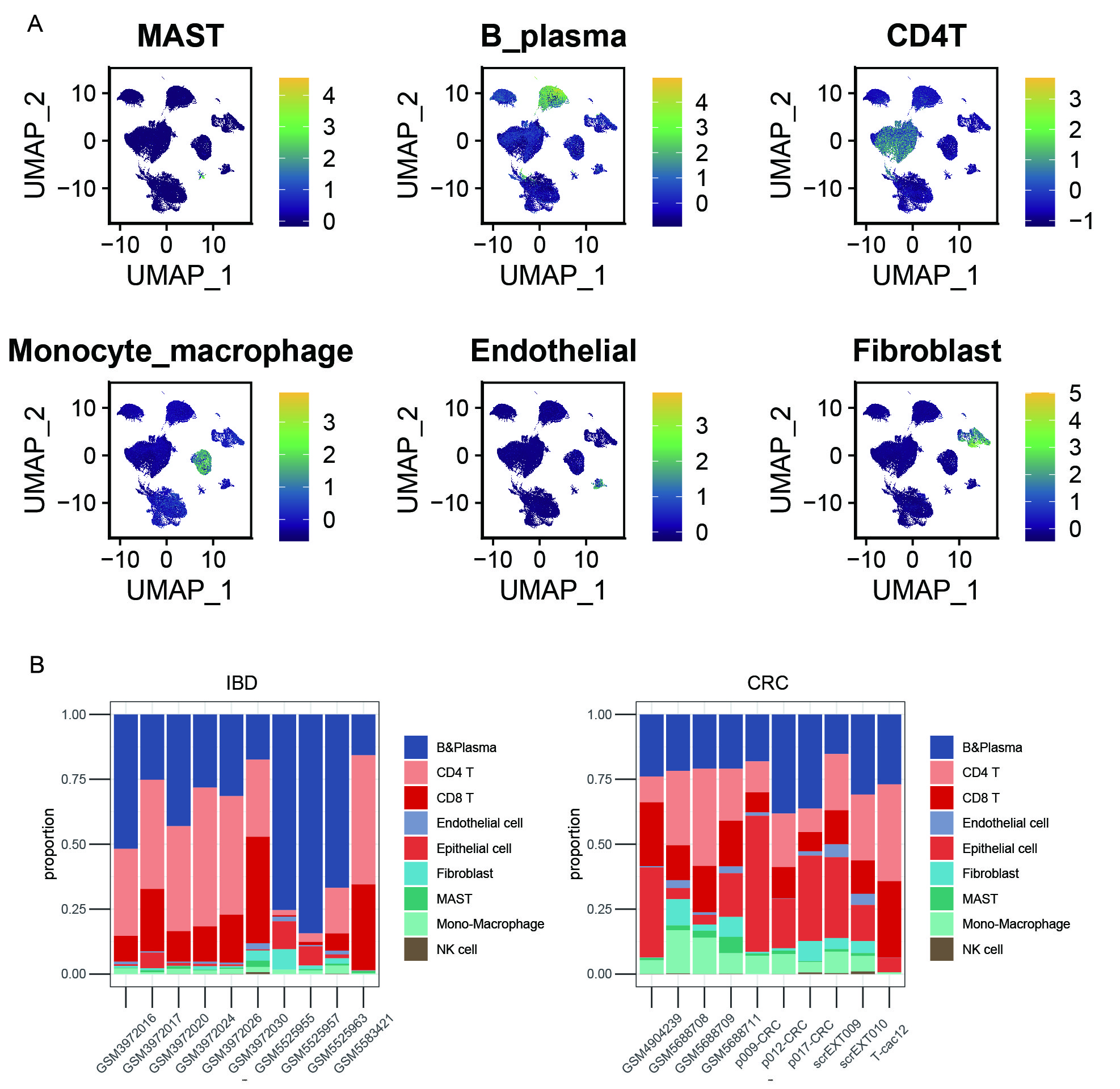

Supplement: Supplementary Figure 1 — Comparison of cell type distribution characteristics. (A) the UMAP plots of gene scores with cell type markers that calculated by function AddModuleScore. (B) The cell proportions of 10 individual samples between IBD and CRC. [file Image_1.jpeg]

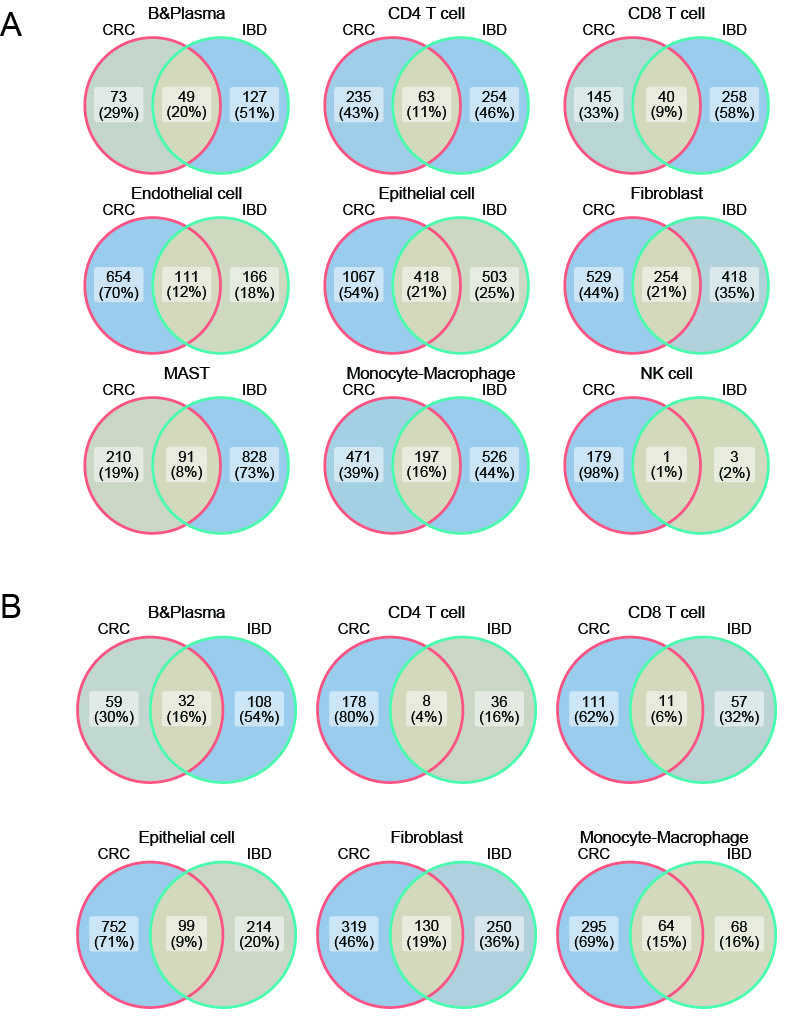

Supplement: Supplementary Figure 2 — Differentially expressed genes (DEGs) of each cell subpopulation in CRC and IBD. (A) Venn diagram of DEGs in nine cell types. (B) Up-regulated DEGs among the six major cell types. [file Image_2.jpeg]

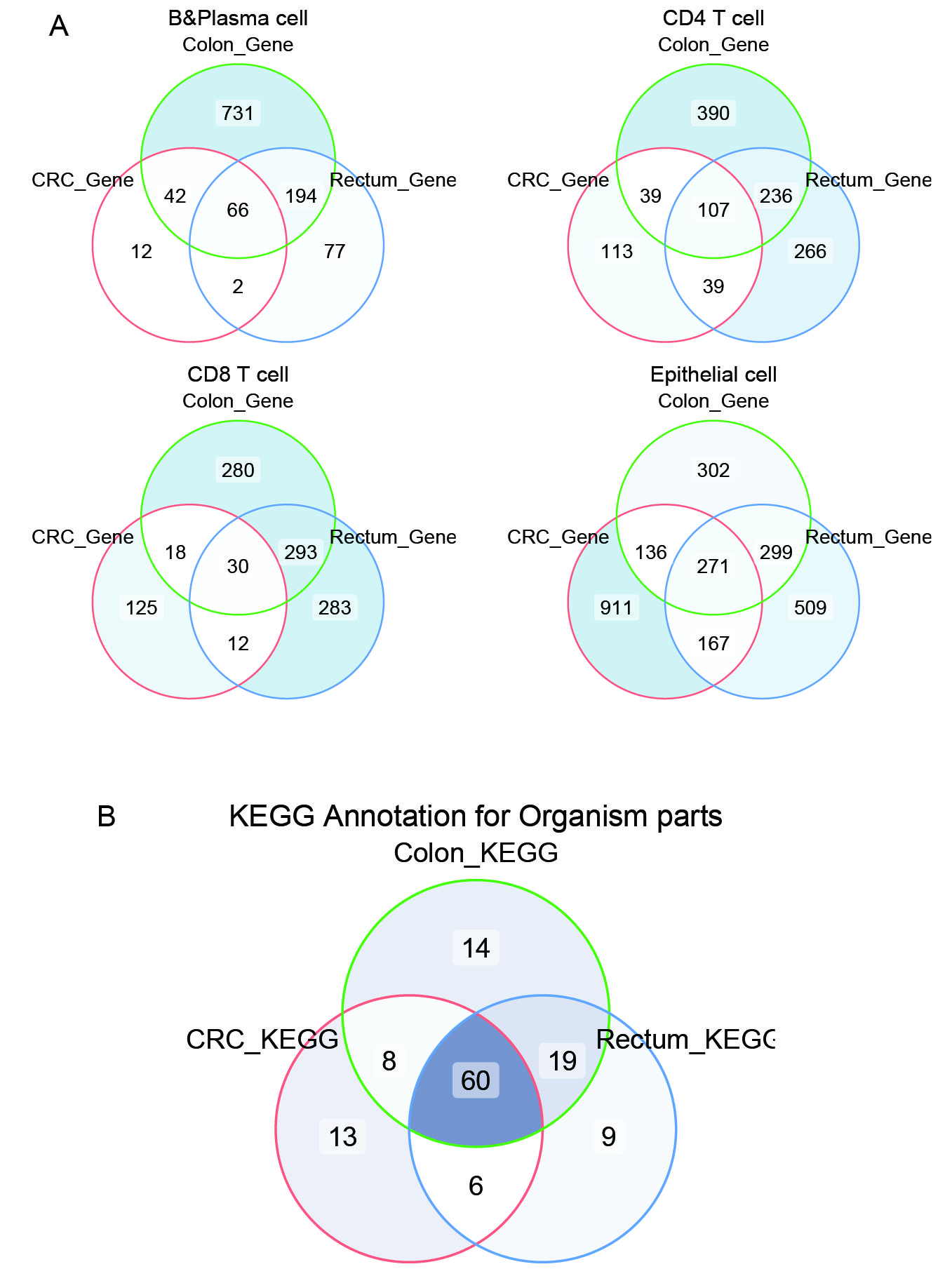

Supplement: Supplementary Figure 3 — The organizational heterogeneity among cell types. (A) Venn diagrams of differentially expressed gene, and (B) Venn diagram of pathway enrichment analysis on epithelial cells, CD4 T cells, CD8 T cells and B cells from the colon and rectum. [file Image_3.jpeg]

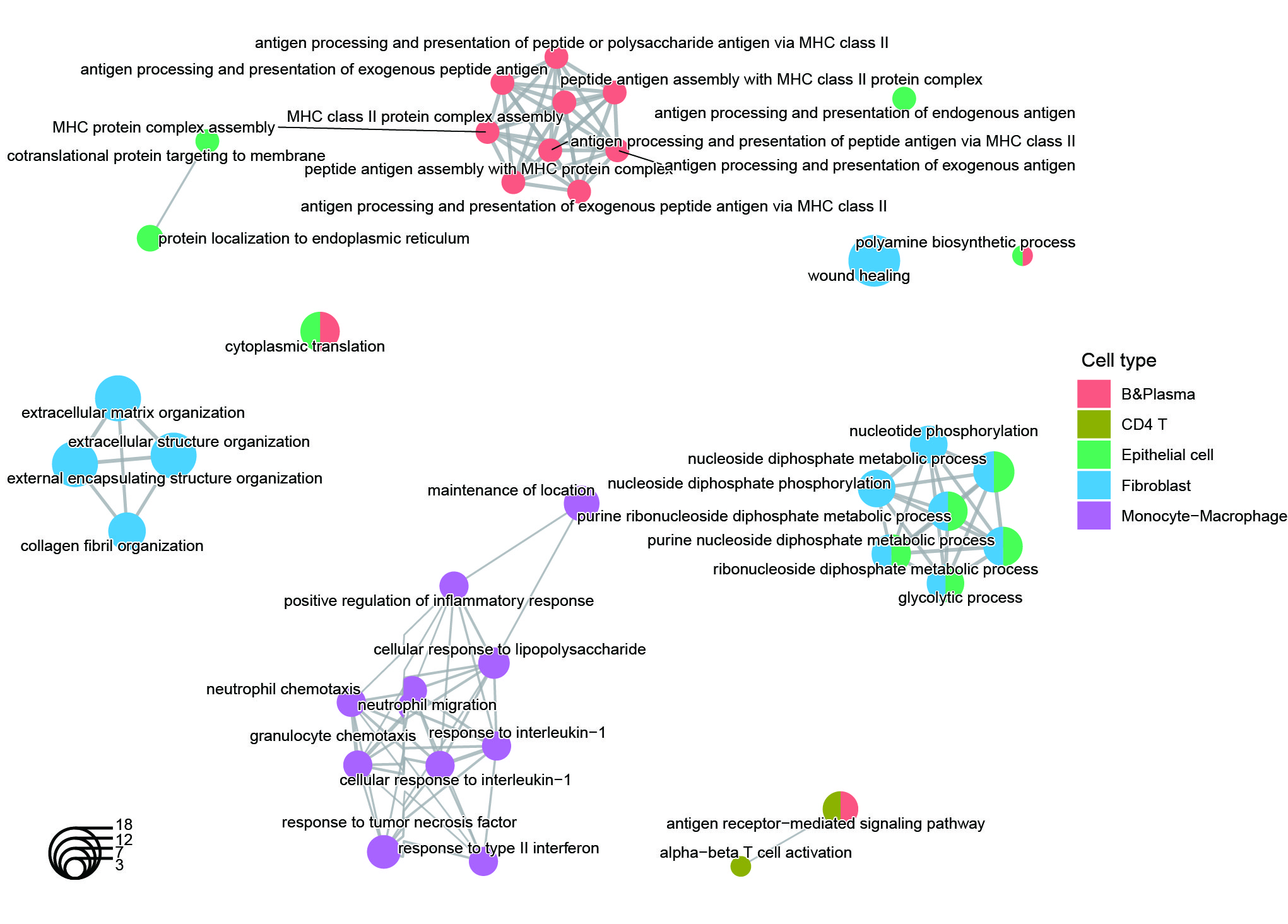

Supplement: Supplementary Figure 4 — GO biological process of the up-regulated differentially expressed genes for nine cell types shared by CRC and IBD. [file Image_4.jpeg]

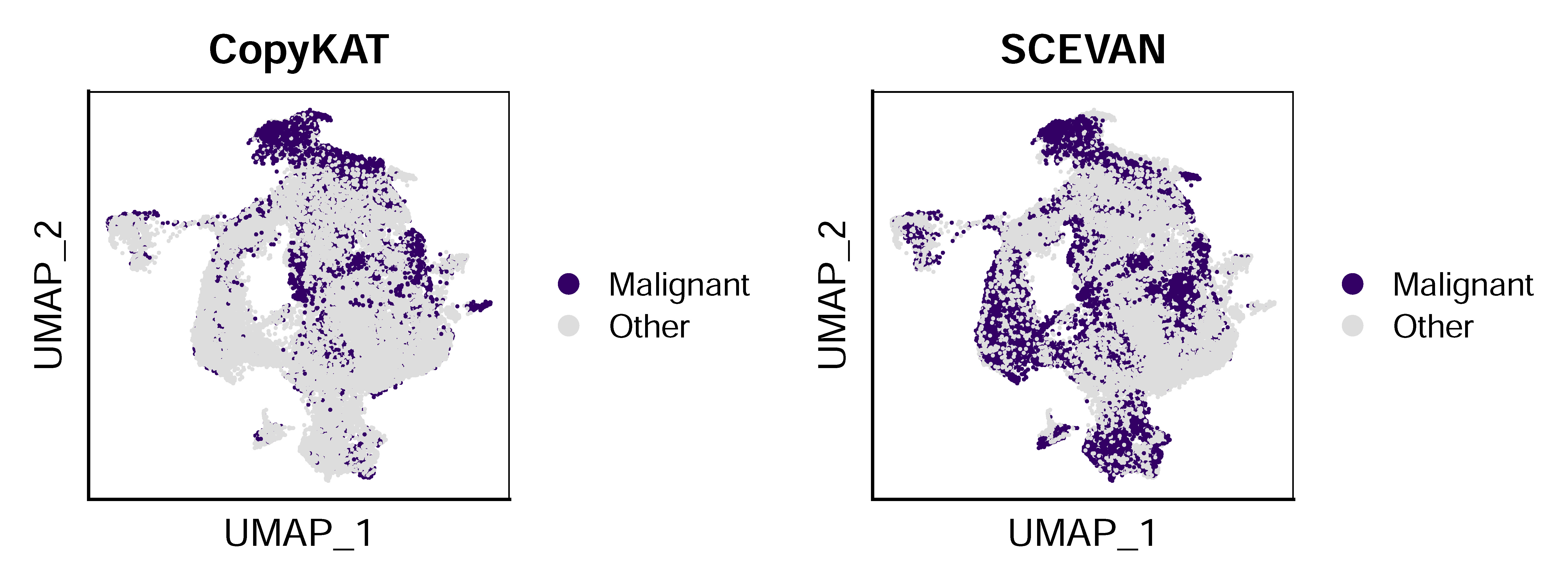

Supplement: Supplementary Figure 5 — UMAP plots of the malignant cells identified by the R packages copykat and SCEVAN. [file Image_5.jpeg]

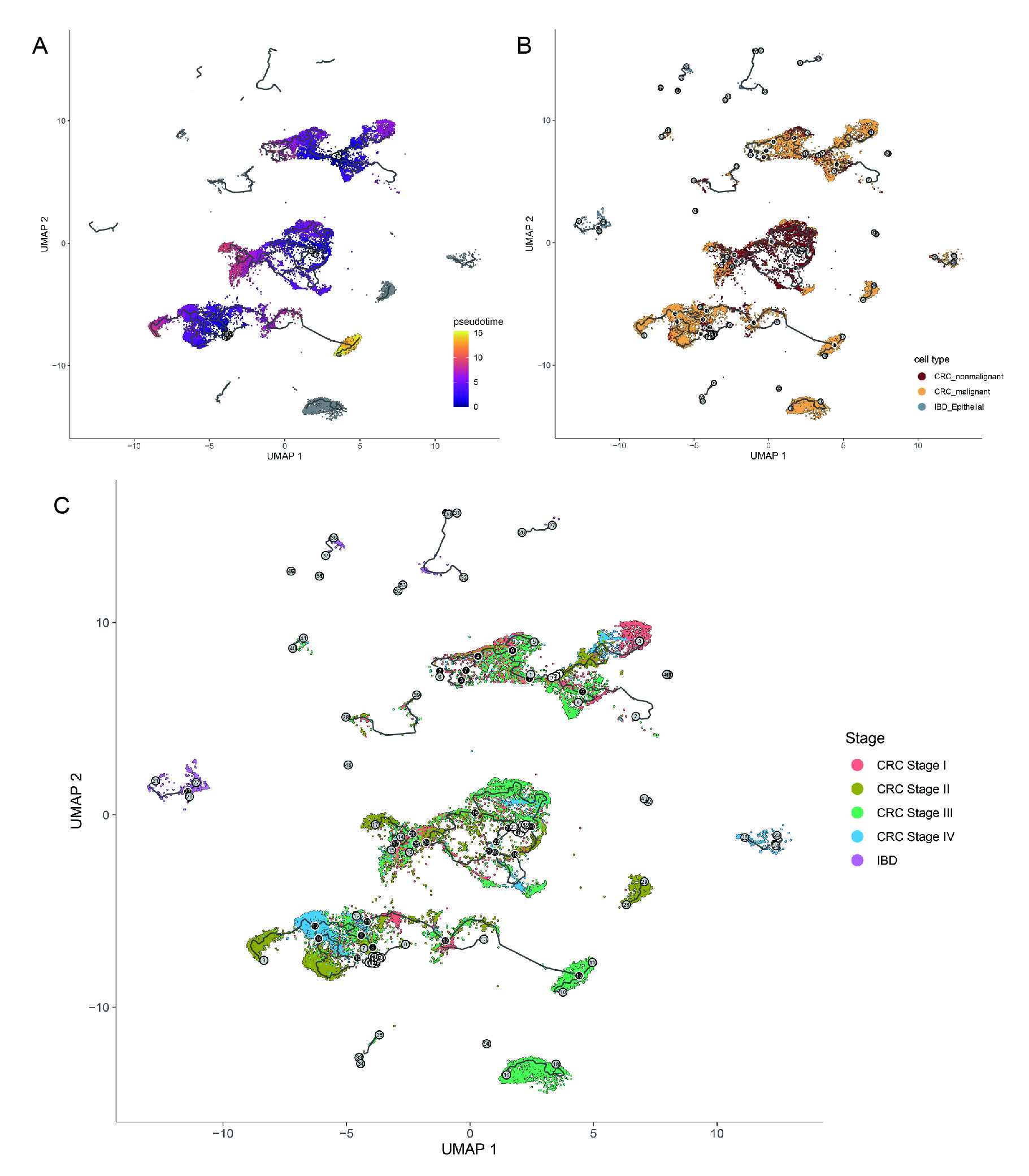

Supplement: Supplementary Figure 6 — the cell trajectory analysis results of IBD epithelial cells, CRC non-malignant cells and malignant cells. (A) The developmental trajectory in the pseudotime analysis. (B) The cell trajectory analysis results of malignant and non-malignant cells. (C) The cell trajectory analysis results of different stages. [file Image_6.jpeg]
